# Egosyntonicity and emotion regulation: a probabilistic model of valence dynamics

**DOI:** 10.1098/rsos.250062

**Published:** 2025-09-24

**Authors:** Eleonora Vitanza, Chiara Mocenni, Pietro De Lellis

**Affiliations:** ^1^ Department of Information Engineering and Mathematics, University of Siena, Via Roma 56, Siena 53100, Italy; ^2^ Department of Electrical Engineering and Information Technology, University of Naples Federico II, Via Claudio 21, Naples 80125, Italy

**Keywords:** emotion dynamics, Markov chains, egosyntonicity

## Abstract

In this paper, we introduce a novel Markovian model that describes the impact of egosyntonicity on emotion dynamics. We focus on the dominant current emotion and describe the time evolution of its valence, modelled as a binary variable, where 0 and 1 correspond to negative and positive valences, respectively. In particular, the one-step transition probabilities will depend on the external events happening in daily life, the attention the individual devotes to such events, and the egosyntonicity, modelled as the agreement between the current valence and the internal mood of the individual. A steady-state analysis shows that, depending on the model parameters, four classes of individuals can be identified. Two classes are somewhat expected, corresponding to individuals spending more (less) time in egosyntonicity experiencing positive valences for longer (shorter) times. Surprisingly, two further classes emerge: the self-deluded individuals, where egosyntonicity is associated to a prevalence of negative valences, and the troubled happy individuals, where egodystonicity is associated to positive valences. These findings are aligned with the literature showing that, even if egosyntonicity typically has a positive impact in the short term, it may not always be beneficial in the long run.

## 1. Introduction

Emotional theories in psychology seek to understand how emotions are generated and experienced, focusing on the interplay between physiological, neurological and cognitive processes, and emotional responses [1]. Emotion dynamical patterns are the complex results of a large number of factors, which shape its evolution over time. We are interested in process theories, where emotions are assumed to be an adaptive element of a feedback loop that involves the situations we encounter, our perceptions of these situations, the emotions they trigger and the actions we take to alter our circumstances [2,3]. In this context, emotional regulation plays a crucial role in human behaviour and well-being. In fact, not only how good or bad people feel on average but also how their feelings fluctuate across time is crucial for psychological health [4]. Emotion regulation can be either deliberate or automatic, the latter being pervasive in everyday life, and having far-reaching consequences for individuals’ emotions [5].

The study of emotion dynamics allowed researchers to make inferences about features of the emotion regulatory system [6]. Namely, the main principles describing emotion dynamics are as follows:

— *Contingency*: emotions are typically contingent on internal or external events. Internal events include physiological states or cognitive processes (e.g. feeling anxious due to personal worries), while external events involve interactions with the environment or social contexts (e.g. feeling happy upon receiving good news). For instance, both excessive and reduced emotional reactivity are believed to contribute to mood disorders such as depression and bipolar disorder.
— *Inertia*: emotional states display an intrinsic resistance to change, as we tend to perceive and interpret the world around us in ways congruent with our current emotional state. For instance, high emotional variability and excessive emotional inertia are both linked to indicators of ill-being and psychopathologies such as depression, bipolar disorder and borderline personality disorder.
— *Regulation*: individuals manage their emotional experiences using various deliberate or automatic strategies. For instance, antecedent-focused regulation involves techniques such as cognitive reappraisal, where a person reinterprets a situation to change its emotional impact, such as seeing a job interview as a learning opportunity instead of a threat. In contrast, response-focused regulation involves managing emotions after they are felt, such as expressive suppression, where one hides feelings of sadness.
— *Interaction*: the components of emotions (physiological, experiential, behavioural), or the emergent emotional states as they are experienced as a whole, continuously interact with, augment and blunt one another, creating a system of interacting elements.

Based on these fundamental principles, several alternative theories on emotions have been developed [7–10]. However, such theoretical frameworks typically lack a clear connection with the substantial amount of empirical research on emotions [11], both within neuroscientific [12] and physiological research [13]. To bridge this gap, researchers have sought to use mathematical models that offer a formal language to capture both quantitative and qualitative aspects of concepts and theories [14–19]. For instance [20], underlines individual differences in affect dynamics through a model that effectively captures the dynamics observed in real data.

Recently, Markov models have emerged as a powerful mathematical tool for capturing the key features of emotion dynamics [21,22]. As argued by Cipresso *et al*. [23], affects, as dynamic and evolving processes, can be effectively analysed using Markov chain approaches to decipher how emotional states change over time. In [24], the authors have used Markov models to capture the mental models that individuals build to predict the others’ emotional dynamics. In [25], two Markov chains have been identified from data on healthy and schizophrenic individuals, respectively, revealing that schizophrenic individuals tend to remain in negative emotional states and show maladaptive transitions.

A recent work from [26] has first used a Markov chain as a generative model of emotions in daily life. In particular, their model, grounded in the principle of contingency, formalized the link between external situations and emotions. Transitions between situations are described by a Markov model, which is characterized by one parameter that corresponds to the probability of transitioning to any of the other situations. In turn, being in a situation determines the mean of the Gaussian distribution that describes the emotions experienced by the individual. This simple, yet effective model is capable of reproducing several empirical phenomena that are observed in the literature, such as the skewed distribution exhibited by some emotion variables [27].

Following [8], Ryan *et al*. [26] also suggest extending their basic model to complete a feedback loop between situations, attention, emotion and action. The goal of our study is then to make a first step in this direction and model the dynamics of the valence, i.e. the pleasantness or unpleasantness associated with an emotion according to the Russell circumplex model [28], illustrating how the conflict between real and expected situations influences emotion regulation. Rather than studying the dynamics of an ensemble of emotions, we focus on the valence of a single generic emotion that represents the overall emergent emotional state at each time instant and build a tractable model that simultaneously accounts for the principles of contingency, inertia and regulation described above. Depending on the model parameters, the valence evolution can be diversely affected by egosyntonicity (i.e. the coherence between the experienced and the expected emotions). Egosyntonicity and egodystonicity describe how certain emotions, ideas and beliefs align or conflict with an individual’s self-perception [29–31]. For example, if a person feels happiness due to upcoming holidays, and such emotion was expected, the experience would be considered egosyntonic. Conversely, if the same person in the same situation expects to feel happiness but instead experiences a different emotion, like distress, it would be regarded as egodystonic.

A parametric study of the model allowed us to identify four key classes of individuals, corresponding to commonly observed healthy or pathological behaviours. Crucially, the four classes differ in the way they regulate the valence based on the time spent in egosyntonicity. Namely, we identify the *balanced happy* (BH), who are healthy individuals that experience long-term positive emotions fostered by egosyntonicity; the *self-deluded* (SD), who are primarily egosyntonic yet experience negative emotions, as in individuals suffering from personality disorders [32]; the *chronically troubled* (CT), who struggle with internal conflict and negative emotions, as in individuals with obsessive compulsive disorder (OCD) [33]; and the *troubled happy* (TH), who maintain a positive attitude despite ongoing internal conflicts, as is typical of self-aware egodystonic individuals who seek help (e.g. through therapy).

The paper proceeds as follows: in §2, we provide a detailed description of the Markovian model, focusing on its defining features and theoretical foundations. In §3, we conduct both a general and a parametric analysis to uncover insights and identify emerging behaviours associated with the model’s dynamics. Finally, §4 concludes the work.

## 2. Modelling emotion dynamics

Following [24] and [26], we employ a discrete-time Markov model to describe the time evolution of the valence of the individual emotional state. We consider three binary state variables associated with the internal mood M, the external events E and the experienced valence V. In particular, a value of 1 (0) for M, E or V represents a positive (negative) mood, external situations and valence, respectively.

The model is grounded in the principles of inertia, contingency and regulation*.*^1^ Specifically, based on the principle of inertia, the individual will have a positive probability r of remaining in the same mood at the next time step. Moreover, based on the principle of contingency, the valence dynamics will be influenced by those of internal mood and external events. Finally, according to the principle of regulation, the transitions between negative and positive valences will be affected by egosyntonicity (i.e. the coincidence between the internal mood and the valence, M=V).

We further assume that the dynamics of the internal mood and external events are not affected by each other, and by the valence. The rationale behind this assumption is to preserve the tractability and interpretability of the model, at the same time retaining its ability to align with the principles of contingency, inertia and regulation. In what follows, we start by describing the dynamics of M and E, to then clarify how they influence the valence dynamics.

### 2.1. Mood and external situations dynamics

The transitions between negative (M=0) and positive (M=1) moods are modelled as a Markov chain with a transition probability matrix:


(2.1) $$\begin{array}{lcr} & \begin{array}{r} P_{M}=\left(\begin{array}{cc} r & 1-r\\ 1-r & r \end{array}\right), \end{array} & \end{array}$$

where the parameter r∈]0,1[ represents the probability of remaining in the same mood in the next time step. The higher r, the more the behaviour of the individual is aligned with the principle of inertia, according to which they are inclined to remain in their habitual mood.

The transitions between negative (E=0) or positive (E=1) external situations are regulated by a Markov chain whose transition probability matrix is


(2.2) $$\begin{array}{lcr} & \begin{array}{r} P_{E}=\left(\begin{array}{cc} s & 1-s\\ 1-s & s \end{array}\right), \end{array} & \end{array}$$

where the parameter s∈]0,1[ denotes the probability of staying in the same external situation in the next time step.^2^

### 2.2. Valence dynamics

The valence of the emotion, representing the individual’s overall emotional state, is formalized by V(t)∈{0,1}*,* and its dynamics is modelled by the transition probability matrix


(2.3) $$\begin{array}{r} P_{V}(t)=\left(\begin{array}{cc} p_{00}^{V}(t) & 1-p_{00}^{V}(t)\\ 1-p_{11}^{V}(t) & p_{11}^{V}(t) \end{array}\right), \end{array}$$

where $p_{ii}^{V}(t)=P(V(t)=i|V(t-1)=i)$ is the probability of remaining in the same valence i at the next time step. The transition probability will depend on whether the individual is egosyntonic or not, i.e. whether its current valence V(t) coincides with its internal mood M(t). In particular,

— the probability of remaining in a negative valence in the next step is
(2.4) $$\begin{array}{rl} p_{00}^{V}(t) & =\omega (1-E(t-1))+(1-\omega )\eta_{0}(t),\\ \eta_{0}(t) & =\left\{ \begin{array}{ll} 1-\alpha , & \text{if }V(t-1)=M(t-1),\\ 1, & \text{if }V(t-1)\neq M(t-1), \end{array}\right. \end{array}$$
  where ω∈]0,1[ represents the attention that magnifies the impact of external events on valence dynamics [34]; for instance, a higher ω implies that positive external events, for which E(t−1)=1, will correspond to lower probabilities of remaining in a negative valence. The time-varying parameter $\eta_{0}$ takes two different values depending on whether the individual is egosyntonic in the previous time step, with α∈]0,1[ modulating how much egosyntonicity decreases the probability of remaining in a negative valence.
— the probability of remaining in a positive valence in the next step is
(2.5) $$\begin{array}{lcr} & \begin{array}{rl} p_{11}^{V}(t) & =\omega E(t-1)+(1-\omega )\eta_{1}(t),\\ \eta_{1}(t) & =\left\{ \begin{array}{cc} 1, & \text{if }V(t-1)=M(t-1),\\ 1-\beta , & \text{if }V(t-1)\neq M(t-1), \end{array}\right. \end{array} & \end{array}$$
  where, as in (2.4), the attention parameter ω quantifies the impact of external events on valence dynamics, whereas $\eta_{1}$, similar to $\eta_{0}$ in (2.4), changes according to the presence or absence of egosyntonicity, with β∈]0,1[ modulating how much egodystonicity decreases the probability of remaining in a positive valence.

We emphasize that we have modelled valence dynamics according to the principles of contingency and regulation. Indeed, the dependence of V on both M and E allows to distinguish between the impacts of internal and external situations on the emotion dynamics, in agreement with the principle of contingency.

In our model, regulation does not take place through deliberate strategies but is mediated through egosyntonicity, which is known to promote acceptance of emotions, in contrast with egodystonic feelings, which often result in avoidance or desire for change [6,35–37]. Specifically, the second term at the right-end side of (2.4) and (2.5) changes depending on whether the individual is egosyntonic, thereby representing an implicit mechanism of adjustment that aligns with the principle of regulation [8]. Egosyntonic experiences are perceived as positive, thus increasing the probabilities of remaining or transitioning towards a positive valence. The opposite happens for egodystonic experiences, which are perceived as unpleasant [33]. Albeit egosyntonicity steers the transition probabilities towards a positive valence, its effect on the long-term, steady-state valence of the emotion is non-trivial and will depend upon the three parameters α, β and ω in (2.4) and (2.5), and r in (2.1), as we will illustrate in §3. The state variables and the related parameters of the model are summarized in table 1.

**Table 1.** State variables and associated parameters modulating their transition probabilities.

state variables	short description	related parameters
M(t)	internal mood	r
E(t)	external situations	s
V(t)	valence of the emotion	α,β,ω

### 2.3. Overall Markov model

By combining equations (2.1)–(2.5), the overall dynamics of the valence, mood and external events yield an eight-state Markov chain, which can be described as follows:

1. A state space X={1,…,8}*,* where 1≡(V=0,M=0,E=0)*,* 2≡(V=0,M=0,E=1)*,* 3≡(V=0,M=1,E=0), 4≡(V=0,M=1,E=1)*,* 5≡(V=1,M=0,E=0)*,* 6≡(V=1,M=0,E=1), 7≡(V=1,M=1,E=0)*,* 8≡(V=1,M=1,E=1). Every possible state corresponds to being in a given valence, mood and subject to a given external situation, simultaneously.
2. An initial state probability vector $\pi_{i}(0)=P[X_{0}=i]$ for all i∈X.
3. The transition probabilities $p_{ij}=P[X_{t+1}=j|X_{t}=i]$, where i is the current state and j is the next state, are collected in the following 8×8 matrix P

(2.6) $$\begin{array}{c} \left(\begin{array}{cccccccc} (\omega +\bar{\alpha }\bar{\omega })rs & (\omega +\bar{\alpha }\bar{\omega })r\bar{s} & (\omega +\bar{\alpha }\bar{\omega })\bar{r}s & (\omega +\bar{\alpha }\bar{\omega })\bar{r}\bar{s} & \alpha \bar{\omega }rs & \alpha \bar{\omega }r\bar{s} & \alpha \bar{\omega }\bar{r}s & \alpha \bar{\omega }\bar{r}\bar{s}\\ \bar{\alpha }\bar{\omega }r\bar{s} & \bar{\alpha }\bar{\omega }rs & \bar{\alpha }\bar{\omega }\bar{r}\bar{s} & \bar{\alpha }\bar{\omega }\bar{r}s & (\omega +\alpha \bar{\omega })r\bar{s} & (\omega +\alpha \bar{\omega })rs & (\omega +\alpha \bar{\omega })\bar{r}\bar{s} & (\omega +\alpha \bar{\omega })\bar{r}s\\ \bar{r}s & \bar{r}\bar{s} & rs & r\bar{s} & 0 & 0 & 0 & 0\\ \bar{\omega }\bar{r}\bar{s} & \bar{\omega }\bar{r}s & \bar{\omega }r\bar{s} & \bar{\omega }rs & \omega \bar{r}\bar{s} & \omega \bar{r}s & \omega r\bar{s} & \omega rs\\ (\omega +\bar{\omega }\beta )rs & (\omega +\bar{\omega }\beta )r\bar{s} & (\omega +\bar{\omega }\beta )\bar{r}s & (\omega +\bar{\omega }\beta )\bar{r}\bar{s} & \bar{\omega }\bar{\beta }rs & \bar{\omega }\bar{\beta }r\bar{s} & \bar{\omega }\bar{\beta }\bar{r}s & \bar{\omega }\bar{\beta }\bar{r}\bar{s}\\ \bar{\omega }\beta r\bar{s} & \bar{\omega }\beta rs & \bar{\omega }\beta \bar{r}\bar{s} & \bar{\omega }\beta \bar{r}s & (\omega +\bar{\omega }\bar{\beta })r\bar{s} & (\omega +\bar{\omega }\bar{\beta })rs & (\omega +\bar{\omega }\bar{\beta })\bar{r}\bar{s} & (\omega +\bar{\omega }\bar{\beta })\bar{r}s\\ \omega \bar{r}s & \omega \bar{r}\bar{s} & \omega rs & \omega r\bar{s} & \bar{\omega }\bar{r}s & \bar{\omega }\bar{r}\bar{s} & \bar{\omega }rs & \bar{\omega }r\bar{s}\\ 0 & 0 & 0 & 0 & \bar{r}\bar{s} & \bar{r}s & r\bar{s} & rs \end{array}\right). \end{array}$$

with $\bar{r}=1-r$, $\bar{s}=1-s$, $\bar{\alpha }=1-\alpha$, $\bar{\beta }=1-\beta$ and $\bar{\omega }=1-\omega$.

The transient dynamics of the model are described by the equation


(2.7) $$\begin{array}{lcr} & \pi (t+1)=\pi (t)P, & \end{array}$$

where π(t) is the (row) probability state vector at time t. Since all the parameters lie in the interval ]0,1[, the Markov chain is finite, irreducible and aperiodic, thus guaranteeing the existence of a unique steady-state probability vector for any choice of the parameters [38]. Therefore, we can study the asymptotic steady-state probabilities $\bar{\pi }=[{\bar{\pi }}_{1},\ldots ,{\bar{\pi }}_{8}]$ of being in any given state. The steady-state distribution $\bar{\pi }$ can then be obtained by solving


(2.8) $$\bar{\pi }=\bar{\pi }P,$$

where the elements of $\bar{\pi }$ satisfy the constraints ${\bar{\pi }}_{j}\geq 0$ and $\sum_{j}{\bar{\pi }}_{j}=1$. We remark that ${\bar{\pi }}_{i}$ can be interpreted as the asymptotic fraction of time spent in a given state. Therefore, in the next section, we analytically and numerically investigate how $\bar{\pi }$ will depend on the model parameters, which are in turn related to behavioural characteristics of individuals.

## 3. Main results

### 3.1. Steady-state probabilities

Here, we compute the steady-state probabilities of (i) having a certain mood, (ii) experiencing a positive/negative external event and iii) experiencing an emotion with positive/negative valence.

We start by focusing on (i) and (ii), since the dynamics of M and E are independent of the valence. By solving the systems ${\bar{\pi }}_{M}={\bar{\pi }}_{M}P_{M}$ and ${\bar{\pi }}_{E}={\bar{\pi }}_{E}P_{E}$, we obtain ${\bar{\pi }}_{M}={\bar{\pi }}_{E}=(0.5,0.5)$*,* i.e. in the long run, the individuals will spend the same amount of time in positive or negative mood states, as well as will equally experience positive and negative external events.

We then turn our attention to (iii) and since the valence depends on both M and E, we solve (2.8) for computing the probability of experiencing a positive emotion. Namely, we compute $\pi_{V_{1}}:=\sum_{i=5}^{8}{\bar{\pi }}_{i}$, thereby obtaining


(3.1) $$\pi_{V_{1}}=\frac{\omega (3\alpha \omega +\beta \omega -\beta -2\omega )(2r-1)/4-\alpha \omega (5r/2-7/4)+(\alpha +\omega )(r-1)}{\omega^{2}(\alpha +\beta -1)(2r-1)-\omega (\alpha +\beta )(3r-2)+(\alpha +\beta +2\omega )(r-1)},$$

whereas $\pi_{V_{0}}:=\sum_{i=1}^{4}{\bar{\pi }}_{i}=1-\pi_{V_{1}}$. This indicates that, in the long run, the steady-state valence probabilities are not influenced by the parameter s that modulates the variability of external events. The analysis of our model suggests that valence dynamics are affected by the attention that we give to external events, rather than by their variability. Indeed, the attention that the individual gives to external events and the mood variability matter, and are captured by ω and r, respectively. The impact of egosyntonicity on $\pi_{V_{1}}$ is evidenced by its dependence on parameters α and β.

We notice that when r=0.5 or α=β, equation (3.1) simplifies to


(3.2) $$\begin{array}{lcr} & \pi_{V_{1}}=\frac{\omega -\alpha (\omega -1)}{2\omega -(\alpha +\beta )(\omega -1)}. & \end{array}$$

In this case, a positive valence is prevalent only if α>β, indicating greater sensitivity to egosyntonicity. Furthermore, we note that when α=β, independent of the value of all other parameters, (3.1) further simplifies, and $\pi_{V_{1}}=0.5$.

### 3.2. Parametric analysis

Here, we numerically investigate the dependence of the steady-state valence probability $\pi_{V_{1}}$ (and, therefore, of the average time spent with a given valence in the long run) on the model parameters. In particular, we vary α,β,r and ω between 0.1 and 0.9 with step 0.1, and we also consider the extreme values 0.01 and 0.99. We fix s=0.1, as external events tend to change rapidly over time, but the results that we are going to illustrate are not affected by the value of s.

Specifically, for each of the 14 641 parameter combinations, we have computed the steady-state valence probability $\pi_{V_{1}}$ from (3.1) and performed a 500 000 steps long simulation of equation (2.7) to compute the difference $\delta_{\mathrm{ego}}$ between the fraction of time steps spent in egosyntonicity and in egodystonicity.

First, we notice that regardless of the values of parameters r and ω, when α>β, the steady-state positive valence probability $\pi_{V_{1}}$ is larger than 0.5, and the opposite happens for α<β, in agreement with the empirical evidence in [33,39] showing that egosyntonic experiences tend to reinforce positive affective states, whereas egodystonic experiences are associated with increased negative affect.

**Figure 1.**
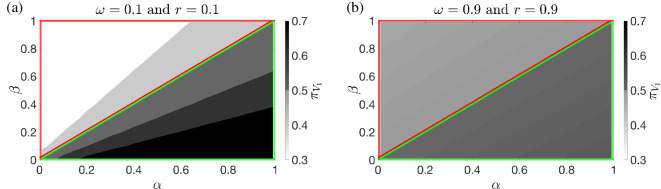
Colour map of the steady-state positive valence probability $\pi_{V_{1}}$ as a function of α and β when (a) ω=0.1, r=0.1 and (b) ω=0.9, r=0.9.

**Figure 2.**
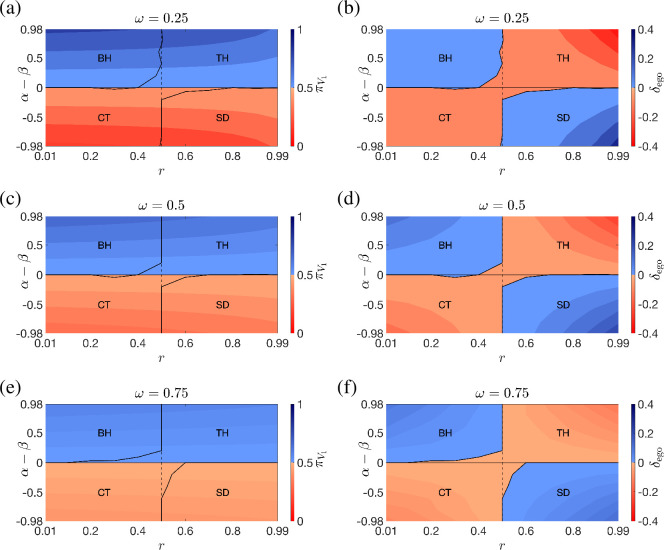
Colour map of the steady-state valence probability $\pi_{V_{1}}$ and the difference $\delta_{\mathrm{ego}}$ between egosyntonicity and egodystonicity frequencies as a function of α−β and r when the attention level is (a),(b) ω=0.25, (c), (d) ω=0.5, (e),(f) ω=0.75. Each plot is divided into four areas, each corresponding to a different class of individuals, where BH stands for balanced happy, TH for troubled happy, CT for chronically troubled and SD for self-deluded. The differences between the four classes are more pronounced for extreme values of α−β and r.

This is illustrated for two sample pairs (r,ω) in figure 1, which shows that, for a given value of α (β), $\pi_{V_{1}}$ monotonically increases with the mismatch α−β, which represents how much an individual is more sensitive to egosyntonicity versus egodystonicity, see the transition probabilities in (2.4) and (2.5). As we have just uncovered the fundamental role played by the mismatch α−β on the asymptotic time spent in a positive valence, we then try to determine how this is related to the time spent in egosyntonicity/egodystonicity. To do this, among all pairs (α,β)*,* we consider those such that α+β=1, so that α−β will range between −0.98 and 0.98. In figure 2, for three representative values of the attention parameter ω, we report two colour maps of the steady-state probability of positive valence $\pi_{V_{1}}(\alpha -\beta ,r)$ and the difference in frequency $\delta_{\mathrm{ego}}(\alpha -\beta ,r)$ between the time spent in egosyntonicity and egodystonicity, respectively. First, by comparing panels (a), (c) and (e), we notice that the effect of an increased mismatch α−β on $\pi_{V_{1}}$ is mitigated by an increase in either the attention to the external events ω or the inertia to mood change r. Moreover, when α=β, $\pi_{V_{1}}=0.5$, as expected from §3. Then, to decipher the relationship between the steady-state probability of positive valence and the time spent in egosyntonicity, we start by comparing $\pi_{V_{1}}(\alpha -\beta ,r)$ and $\delta_{\mathrm{ego}}(\alpha -\beta ,r)$ when ω=0.5, see panels (c) and (d). We notice the emergence of four classes of individuals, characterized by qualitatively different behaviours:

(i) The balanced happy (BH) are individuals who benefit from the larger share of time spent in egosyntonicity ($\delta_{\mathrm{ego}}>0$) and experience positive valences with a probability higher than chance ($\pi_{V_{1}}>0.5$). In figure 2, BH are encountered for parameter combinations corresponding to blue regions both in the left panels and in the corresponding right panels.
(ii) The self-deluded (SD) prevalently experience negative emotions ($\pi_{V_{1}}<0.5$), albeit being generally in sync with themselves ($\delta_{\mathrm{ego}}>0$). In figure 2, SD are encountered for parameter combinations corresponding to coral regions in the left panels and blue regions in the corresponding right panels.
(iii) The chronically troubled (CT) are individuals who pay the price for being prevalently egodystonic ($\delta_{\mathrm{ego}}<0$) by experiencing negative emotions ($\pi_{V_{1}}<0.5$). In figure 2, CT are encountered for parameter combinations corresponding to coral regions both in the left panels and in the corresponding right panels.
(iv) The troubled happy (TH) prevalently experience positive emotions ($\pi_{V_{1}}>0.5$) despite spending more time in egodystonicity ($\delta_{\mathrm{ego}}<0$). In figure 2, TH are encountered for parameter combinations corresponding to blue regions in the left panels and coral regions in the corresponding right panels.

Looking at panels (a), (b), (e) and (f), we notice that the above classification holds for all values of ω. The effect of variations in the attention parameter is that of mitigating or amplifying the difference between the classes.

## 4. Discussion

In this work, we introduced a novel Markovian model of emotion dynamics that is grounded on three fundamental principles of the branch of psychology that studies how emotions shape over time: inertia, contingency and regulation. In particular, the principle of inertia is captured by the internal mood dynamics, with a parameter that quantifies the reluctance to change mood. The principle of contingency is reflected in the valence dynamics, which depends on the complex interplay between the internal mood and the external events. Finally, the model also considers an implicit mechanism of regulation, whereby the individual’s behaviour modifies depending on whether they are egosyntonic.

By performing a parametric analysis of the model, four classes of individuals have emerged based on the relationship between the time spent in egosyntonicity and the long-term emotional valence: the BH, the SD, the CT and the TH. Interestingly, the four identified classes correspond to behaviours that are typically observed either in healthy individuals or in individuals with mental disorders.

The class of BH includes individuals who spend more time in egosyntonicity, thus living in harmony with their emotions. This healthy behaviour enhances their mental health by increasing the experienced positive emotions. On the opposite end, the CT is often in internal conflict and experiences negative emotions in the long term. Such a behaviour is typical, for instance, of obsessive compulsive disorder (OCD) and eating disorder (ED). Indeed, obsessions in OCD and intrusive thoughts in ED are generally egodystonic, as they are experienced as distressing and unwanted [33]. More generally, the experience of egodystonic thoughts or emotions is not uncommon. Most people will note strange, out-of-character thoughts at some point. But for someone, the fear, anxiety and self-judgement associated with the experience of these egodystonic thoughts can negatively impact emotional well-being and may be associated with symptoms [30].

The remaining two classes, the SD and TH, are somewhat unexpected, yet very interesting. Indeed, from the valence dynamics in §2, one could inaccurately infer that egosyntonicity may only have a positive impact on the valence. However, the interplay with mood dynamics makes this relationship less obvious. For instance, the class of SD includes individuals who are generally in sync with themselves but experience negative emotions in the long term. They can be viewed as individuals who ignore signs of distress, living with a false sense of well-being that does not materialize into lasting happiness. Such behaviour is typical of personality disorders. For instance, grandiosity is an egosyntonic symptom of the narcissistic personality disorder [32], which strongly challenges the treatment of the disorder, as the individual may be reluctant to change their behaviour.

On the other hand, the TH, despite often being in internal conflict (egodystonic), manages to maintain a positive emotional valence in the long term. Their discomfort seems to drive growth, leading to unexpected emotional resilience. This is the paradigmatic behaviour observed in a successful therapy, where individuals might deliberately engage in egodystonic behaviours for a period of time with the goal of feeling better in the long term. In such individuals, therapy helps them navigate internal conflicts and fosters personal growth.

The behavioural repertoire showcased by our minimalistic model is surprisingly rich, and yet grounded on fundamental psychological principles.

### 4.1. Limitations and future work

The promising results of this work are, however, not free of limitations and could pave the way for future investigations.

First, the binary modelling of mood, valence and external events could be replaced by a multi-level discretization that would enable a more nuanced description of their dynamics. Second, the model deliberately focuses on a single emotion, which can be considered the prevalent one that the individual is experiencing. This allows to elucidate the relationship between egosyntonicity and behavioural regulation; however, future, richer models should consider the effect of interacting emotions. Indeed, in its current form, the model captures the dynamics of the dominant emotion and simply assumes mood dynamics to be independent from other variables. To represent moods that arise from the combination of different emotions, mood dynamics should then be changed, considering transition probabilities that are a function of the different emotions experienced by an individual at each time step. Third, albeit the model reproduces four well-known interplays between egosyntonicity and emotional well-being, its present formulation does not allow individuals to learn and evolve from past experiences. Future work could consider the presence of adaptive mechanisms, whereby the individual learns from past experiences and modifies their behavioural parameters. This would turn the model parameters into manipulable variables that can be adjusted, for instance, through psychological therapy. Finally, although our model is founded on psychological principles and is capable of reproducing commonly observed behaviours, future empirical works could collect longitudinal data on emotion dynamics with the goal of further refining the model and the underlying hypotheses on which it is based. For instance, the possible dependencies between internal mood and external events could be incorporated in the model, also including feedback mechanisms from valence to internal and external states.

## Data Availability

This article has no additional data.

## Footnotes

^1^
We do not consider the principle of interaction since we are focusing on the dominant emotion.

^2^
To exclude the case of a deterministic evolution of *M* and *E*, we have considered both *r* and *s* in the open interval ]0,1[.
